# Self-Reported Health in Patients on or at Risk of Sick Leave Due to Depression and Anxiety: Validity of the EQ-5D

**DOI:** 10.3389/fpsyg.2021.655151

**Published:** 2021-10-28

**Authors:** Kenneth Sandin, Gemma E. Shields, Ragne G. H. Gjengedal, Kåre Osnes, Marianne Tranberg Bjørndal, Odin Hjemdal

**Affiliations:** ^1^Division of Mental Health and Substance Abuse, Diakonhjemmet Hospital, Oslo, Norway; ^2^Department of Psychology, Norwegian University of Science and Technology, Trondheim, Norway; ^3^Manchester Centre for Health Economics, Division of Psychology and Mental Health, Faculty of Biology, Medicine and Health, School of Health Sciences, University of Manchester, Manchester, United Kingdom

**Keywords:** depression, anxiety, sick leave, EQ-5D, validity, self-reported health

## Abstract

**Objectives:** The EQ-5D is a generic, self-report measure of health that is increasingly used in clinical settings, including mental health. The EQ-5D captures health using five dimensions: Mobility, Self-care, Usual activities, Pain/discomfort, and Anxiety/Depression. The validity of the EQ-5D is previously unexplored in patients on or at risk of sick leave due to depression and anxiety. The study’s aim was to examine its validity in this group of patients.

**Methods:** Baseline data were collected from self-report questionnaires in an observational study (*N*=890) at a Norwegian outpatient-clinic. Participants were adults on or at risk of sick leave due to depression and anxiety who were referred for treatment by general practitioners. The crosswalk methodology was applied to estimate the EQ-5D value. Validity was assessed by comparing responses on the EQ-5D with the Beck Depression Inventory-II (BDI-II), the Beck Anxiety Inventory (BAI), and Subjective Health Complaints (SHC). An ordinal regression model was used to assess known-groups validity. Convergent validity was assessed using Pearson’s correlation coefficient, and a multivariate regression model that included sociodemographic characteristics.

**Results:** The mean EQ-5D value was 0.631, indicating reduced health status compared to “full health” anchored at 1.0, and patients reported moderate levels of depression and anxiety. Ordinal regression indicated that the EQ-5D could discriminate between different levels of symptom severity for depression and anxiety. The EQ-5D value showed significant correlation with the clinical measures; *r*=−0.52 for the BDI-II, *r*=−0.49 for the BAI, and *r*=−0.44 for SHC. The multivariate regression showed that the clinical variables significantly predicted the EQ-5D value, explaining 40.1% of the variance. Depression and anxiety scores were the largest determinants of EQ-5D value, respectively, whilst sick leave, subjective health complaints, and gender made moderate contributions.

**Conclusion:** The EQ-5D showed indication of validity in patients on or at risk of sick leave due to depression and anxiety in the present study. The EQ-5D value was sensitive to both symptom severity and functional impairment in the form of sick leave. The findings support the EQ-5D as a feasible and relevant measure of health status in these patients.

## Introduction

Common mental disorders such as depression and anxiety are frequently comorbid, and affect a fifth of the working population at any given time (Lamers et al., 2011; OECD, 2015a). Functional impairment is a key feature of these disorders, which may partially be related to typical symptoms like withdrawal and isolation (OECD, 2015b). Globally, mental illness is a leading cause of disease burden, estimated to account for 32.4% of all years lived with disability (Vigo et al., 2016). Across the EU region mental ill health costs in excess of € 600 billion per year (4.4% of GDP), and the majority of the cost comes from lost productivity through sick leave and disability (OECD/EU, 2018). Employment rates among people with depression and anxiety are 10–15% lower than for the general population (Norstrom et al., 2019). Loss of employment leads to worse health, including an increase in all-cause mortality (Voss et al., 2004), highlighting the impact of these disorders on wider health status.

The cost of mental health problems for individuals and society has led to calls for increased funding for mental health care (Chisholm et al., 2016). But any increase in investment in mental health must be weighed against potential gains of investing in other areas of health. This inherent dilemma of health care prioritisation has led to a growing interest in instruments that can help compare disease burden across patient groups (Drummond et al., 2015). Generic measures of health can help facilitate such comparisons, for instance through generating quality-adjusted life-years (QALYs) used in cost-effectiveness analyses (Devlin et al., 2020).

The most widely used generic measure of patient-reported health is the EQ-5D (Devlin et al., 2020). The instrument was initially developed by an interdisciplinary group with the aim of measuring and valuing health states (Devlin and Brooks, 2017). Expert reviews of existing literature and empirical testing resulted in the publication of a self-report questionnaire that recorded health across five dimensions: Mobility, Self-care, Usual activities, Pain/discomfort, and Anxiety/Depression. These five dimensions were rated on a three-level severity scale from “No problems” to “Moderate problems” to “Extreme problems” (EuroQol, 1990). The EQ-5D has since seen increasing use in clinical research, and its use in appraising health care interventions is recommended by bodies such as the National Institute of Health and Care Excellence in the United Kingdom and the National Institute of Public Health in Norway (NICE, 2018; NIPH, 2019).

Substantial use of the three-level version of the EQ-5D has since led to concerns that the instrument has limited range in capturing variation in health. Studies on both general and clinical populations showed that health problems were not adequately measured, for instance through pronounced ceiling effects (Herdman et al., 2011). This was also the case for mental health populations: reasonable validity was seen in depression, whilst for anxiety disorders the results were more mixed (Sonntag et al., 2013; Brazier et al., 2014). Given the variable performance of the three-level EQ-5D across multiple patient groups, a new version of the EQ-5D, containing five levels of severity, was developed to improve the instruments measurement characteristics (Herdman et al., 2011). Evidence on the validity of the new five-level version is so far limited, and there is thus a need for studies investigating its validity across different patient groups (Mulhern et al., 2014), including mental health patients (Brazier et al., 2014).

To be a valid measure of self-reported health for patients with depression and anxiety, the EQ-5D would need to adequately reflect the wide impact that these disorders have on health. In addition to symptom severity, reduced functioning is a key feature of these disorders (Chevance et al., 2020). This is supported by the high prevalence of sick leave and disability seen among people with depression and anxiety (Norstrom et al., 2019). For this reason, increasing attention is given to work status and sick leave in studies of interventions for depression and anxiety (Cullen et al., 2018; Salomonsson et al., 2018). There is now broad agreement on the importance of helping these patients avoid sick leave, and that success of interventions should also be measured in terms of maintaining employment or returning to work (OECD, 2012). Sensitivity to functional impairment such as sick leave would thus support the validity of the EQ-5D for this patient group, and its usefulness for evaluating interventions.

Research on the previous three-level version of the EQ-5D showed some indication of ability to capture functional impairment in depression and anxiety. One study found that patients with depression in primary care had substantially lower health status as recorded by the EQ-5D. Furthermore, patients in the sample who were on sick leave reported a 10% lower EQ-5D value compared to those who were not on sick leave (Sobocki et al., 2007). A study that used a random sample of 43,589 individuals from the general Swedish population found that sick leave was associated with more problems reported on the three-level EQ-5D (Eriksson et al., 2008). Another Swedish study showed that lower EQ-5D scores predicted an increase in sick leave in patients with musculoskeletal complaints (Stigmar et al., 2013). In Norway, a randomised controlled trial found significantly reduced health status in patients with common mental disorders and work-impairment (Reme et al., 2015).

In addition to symptom severity and reduced functioning, overall health status may also be affected by sociodemographic factors such as age, gender, marital status, and level of education (Braveman and Gottlieb, 2014). These sociodemographic factors have also been shown to be associated with sick leave (Mastekaasa and Melsom, 2014; de Vries et al., 2018). The degree to which these factors impact the health status of patients with depression and anxiety could thus also help shed light on the instrument’s validity.

The sensitivity of the five-level version to depression, anxiety, and functional impairment in the form of sick leave has yet to be investigated. Therefore, the aim of the present study was to help address this gap by exploring the construct validity of the EQ-5D for patients on or at risk of sick leave due to depression and/or anxiety. Construct validity is the degree to which an instrument measures the intended construct (Piedmont, 2014). Two types of construct validity were examined: known-groups and convergent validity. Known-groups validity indicates that an instrument should be able to discriminate between groups known to differ on the variable of interest (Davidson, 2014). Convergent validity indicates that two instruments that measure related constructs should be highly correlated (Chin and Yao, 2014). To assess the validity of the EQ-5D on these counts, the associations with condition-specific measures of depression and anxiety were assessed.

The current study investigated the following hypotheses: that known-groups validity was supported by (1a) patients on or at risk of sick leave due to depression and/or anxiety reporting reduced health status on the EQ-5D compared to the general population norms, and (1b) that the EQ-5D was able to distinguish between patient groups with different levels of depression and anxiety severity. Additionally, that convergent validity was supported by (2a) the EQ-5D showing significant negative correlations with symptom-specific measures, and (2b) health status recorded by the EQ-5D was significantly explained severity of depression and anxiety symptoms, and by sick leave.

## Materials and Methods

### Study Context and Participant Characteristics

Data were collected in a naturalistic observational study at an outpatient clinic at Diakonhjemmet Hospital in Oslo, Norway. The clinic is part of the national specialised mental health care services. This observational study is part of the project “The Norwegian studies of psychological treatment and work (NOR-WORK).” The treatment at the clinic consists of either Metacognitive therapy (MCT) or Cognitive behavioural therapy (CBT), paired with work-focused interventions. The work-focused interventions are aimed at either helping patients remain at work, or in the case of sick leave, return to work (Gjengedal et al., 2020).

The patients who participated in the study were initially referred by their general practitioners for treatment of depression and/or anxiety. At the clinic, patients are initially screened by clinical psychologist for treatment eligibility according to clinical and diagnostic criteria, including by use of the Mini-International Neuropsychiatric Interview (MINI; Lecrubier et al., 1997). As the clinic offers work-focused treatment, the target population consists of patients on or at risk of sick leave due to depression and/or anxiety. That the patients conform to these criteria is firstly assessed through the referral done by the general practitioner, which is evaluated by a clinical psychologist. A second clinician then sees the patient for an assessment session, determining in cooperation with the patient that the patient has clinically relevant symptoms of depression and anxiety, and is experiencing work-related difficulties that could benefit from work-focused treatment. Patients thus had to be adults of working age (age 18–70years) to participate in the study. Patients were not included in the study if they were suffering from severe mental illness such as bipolar disorder or other psychotic disorders, if they were considered to be at high risk of suicide, or if they were engaging in active substance abuse, or suffered from cluster A or B personality disorder. All patients gave written, informed consent before participation in the study. Data were collected from May 2017 through December 2019, and 890 patients fulfilled the inclusion criteria and consented to participate in the study.

### Ethical Considerations

The study is classified as health service research under Norwegian regulation. The Norwegian Data Protection Agency has designated that treatment providers (i.e., hospitals) are responsible for proper data management in such cases. As the information being collected is part of ongoing provision of health care, no further approval is needed beyond consent from the individual patient. Written consent was obtained from all participants. Data collection and security in the present study was managed by Diakonhjemmet Hospital, and approval of data handling was granted by Oslo University Hospital, approval number 2015/15606. The study was carried out in accordance with the principles of the Helsinki declaration.

### Measures

Clinical and sociodemographic data were collected from patient journals and from self-report questionnaires filled in by patients at the clinic.

#### EQ-5D

The EQ-5D questionnaire measures health status using five dimensions (Mobility, Self-care, Usual activities, Pain/discomfort, and Anxiety/depression). Designed to improve upon the three-level version, The EQ-5D-5L scores each dimension on five levels of severity ranging from 1=“No problems” to 5=“Extreme problems” (Herdman et al., 2011). For example, on the Anxiety/depression dimension, patients report their responses from 1 (“I am not anxious or depressed”) to 5 (“I am extremely anxious or depressed”). The responses on the five dimensions yield the EQ-5D profile, e.g., “11,111” in the case of “No problems” on all dimensions, or “55,555” in the case of “Extreme problems” on all dimensions. There are 3125 (5^5^) possible EQ-5D profiles in the five-level version (Devlin et al., 2020).

These health profiles can in turn be converted into a single EQ-5D value using preference based weights. Value sets (or tariffs) are available to support the calculation of the EQ-5D values (Devlin et al., 2020). A study is underway to acquire a value set for Norway, but this is not yet available (NIPH, 2019; Moen Hansen et al., 2020). In such cases it is recommended to use a crosswalk (or mapping) system (NICE, 2019), and this crosswalk system was used in a recent study obtaining Norwegian EQ-5D population norms (Garratt et al., 2021). The same crosswalk methodology was used in the present study when calculating the EQ-5D value. Although negative values are possible, the EQ-5D value ordinarily ranges from 0, which represents death, to 1 which represents full health. A score of 1.000 (i.e., full health) corresponds to a health profile of “11,111,” i.e., reporting “No problems” across all dimensions. Healthy populations typically report EQ-5D values close to 1; for instance, the study obtaining data from the Norwegian general population found a mean value of 0.805 in a postal survey (Garratt et al., 2021). Note also that when reporting the EQ-5D values it is common to use three decimals (Devlin et al., 2020).

In addition to the EQ-5D profile and the EQ-5D value, the EQ-5D also contains a visual-analogue scale of health, the EQ visual analogue scale (VAS). On the EQ VAS, patients indicate their subjective health state on a visual barometer from a minimum of 0=worst imaginable health, to a maximum of 100=best imaginable health (Herdman et al., 2011).

#### Anxiety

The Beck Anxiety Inventory (BAI) is a self-report measure of anxiety severity over the last week. Examples of items in the BAI are “Heart pounding or racing” and feeling “Nervous.” The BAI has 21 such items where these symptoms of anxiety are scored on a scale of severity ranging from 0 to 3, giving total score ranging from 0 to 63. Higher scores indicate more severe symptoms. Recommended scoring of the BAI suggests that 0–15 indicate minimal symptoms, 16–25 moderate symptoms, and 26–63 severe symptoms. In literature reviews, the BAI has shown high internal consistency with an alpha of 0.92 and a test-retest reliability of 0.75 (Beck et al., 1988). In the current study, we report the Omega as this may be a more precise measurement (Peters, 2014). The Omega of the BAI in this study was 0.90.

#### Depression

The Beck Depression Inventory-II (BDI-II) is a 21 item self-report measure of depression symptom severity over the last 2weeks. As with the BAI, the BDI has 21 items that are scored on a severity scale ranging from 0 to 3, giving a score range of 0–63. Higher score indicates more severe symptoms (Beck et al., 1996). As an example, the first item asks patients to rate their sadness from 0 (“I do not feel sad”) to 3 (“I am so sad or unhappy that I cannot stand it”). A BDI-II score of 0–13 indicates minimal symptoms, 14–19 mild symptoms, 20–28 moderate symptoms, and 29–63 severe symptoms. A review of the literature indicates that the BDI-II is psychometrically sound with internal consistency showing an alpha around 0.90, and a test-retest reliability ranging from 0.73 to 0.96 (Wang and Gorenstein, 2013). In the current study, we found the Omega to be 0.86.

#### Subjective Health Complaints

The subjective health complaint (SHC) is a self-report questionnaire that contains 29 items measuring subjective health complaints along five factors: musculoskeletal pain, pseudo-neurology, gastrointestinal problems, allergy, and flu. For example, patients are asked to rate pain in arms, leg, or lower back. The aim of the SHC is to provide a simple measure of the most common complaints seen by general practitioners while “avoiding diagnoses and theoretical bias.” The severity of complaints on each item is rated on a four point Likert-scale from 0 (no complaints) to 3 (severe complaints) during the last 30days. The total score of the scale thus ranges from 0 to 87 where higher score indicates worse complaints. Factor analysis of the questionnaire has shown that the greatest proportion of variance of scores is explained by musculoskeletal pain (Eriksen et al., 1999). This measure of subjective health complaints was included as depression and anxiety both have well-known comorbidity with musculoskeletal pain (Bair et al., 2003; Asmundson and Katz, 2009). In the current study, the Omega for the SHC was 0.82.

#### Sick Leave

Sick leave in the present study was collected from patients *via* self-report questionnaires. For the purpose of the study, we encoded sick leave as a dichotomous variable where patients who were fully working with no social benefits of any kind were coded as “0,” and patients on sick leave were coded as “1.” We did not collect data on degree of sick leave (e.g., whether a patient was on 100 or 50% sick leave).

#### Sociodemographic Variables

We included age, gender, cohabitation, and level of education in the analyses to measure relevant sociodemographic aspects of health. Cohabitation was dichotomised as living with partner or living alone. Education level was included as a dichotomous variable, those without higher education were coded as 0, and those with higher education were coded as 1. “Higher education” in this regard refers to any completed degree beyond upper secondary school, i.e., the first 12years of schooling.

### Statistical Analyses

All analyses were carried out using STATA 16.1 (StataCorp, 2019). Assessment of missing data found low incidences throughout the measures. The BDI-II, the BAI, and the EQ-5D, <2% on all items. The SHC had <5% missing on all items. Little’s MCAR test was not significant for our dependant variable, the EQ-5D value (*χ*^2^ 19.69, DF=13, *p*=0.103). This indicates that these values were missing completely at random. Little’s MCAR test was significant for the BAI (*χ*^2^ 1113.19, DF=1,040, *p*=0.006), the BDI-II (*χ*^2^ 704.38, DF=628, *p*=0.018), and SHC (*χ*^2^ 1918.09, DF=1,566, *p*<0.001), indicating that these variables were not missing completely at random. Further exploration of missing patterns in the BAI, the BDI-II, and the SHC showed that missing data were explained by the covariate “education,” i.e., patients with higher education were more likely to return complete forms. Guidance on EQ-5D data states that general methods used for handling missing data also apply to the EQ-5D (Devlin et al., 2020). Recent guidelines indicate that, as a rule of thumb, it may be a valid approach to ignore missing data if missingness is below 5% (Jakobsen et al., 2017). Although this was the case in the present study, we chose to replace missing data on individual items by weighted means. This method was developed for handling missing data in patients with depression and has shown good precision when used with this patient population (Gale and Hawley, 2001). Data were tested for normality and the clinical variables were found to be within the acceptable range for use of parametric tests as skewness and kurtosis were within −1 to +1 on all measures (Hair et al., 2017).

We defined floor effect for the EQ-5D as proportion of patients reporting “No problems” on all dimensions (i.e., an EQ-5D profile of “11,111”). We defined ceiling effect of the EQ-5D as reporting “Extreme problems” on all dimensions (i.e., an EQ-5D health profile of “55,555”). For the BDI-II, the BAI, and the SHC, floor and ceiling were defined as patients reporting either the lowest or highest possible sum score, that is 0 or 63 for the BAI and the BD-II, and 0 or 87 for the SHC.

It is recommended to present EQ-5D scores with descriptive statistics before presenting any further findings (Devlin et al., 2020). Therefore, we report the proportion of patients that indicated each level of severity for each dimension of the EQ-5D. We also present the mean EQ-5D values and EQ VAS scores by groups based on clinical and sociodemographic characteristics. We then compared the proportion of patients reporting “No problems” to patients reporting any other levels of severity (Devlin et al., 2020). Using the recently published Norwegian population norms (Garratt et al., 2021), we explore known-groups validity by comparing the patients in our study and participants in the general population study who reported “no problems” vs. all other levels of severity. For known-group validity within the sample, we divided the patients into quartiles based on severity of depression and anxiety symptoms as recorded by the BDI-II and BAI scores. Test of Cuzick (1985) for trends, which is a Wilcoxon rank-sum type test for three groups or more, was used to examine if the EQ-5D utility could distinguish between the groups. For the EQ-5D dimensions, we performed an ordinal logistic regression. The severity groups divided by quartiles was used as the dependant variable, and the EQ-5D dimensions were used as predictor variables. The model was tested for multicollinearity. No predictor variable had a variance inflation factor (VIF) higher than 1.38, indicating that multicollinearity was not a problem.

We then explored convergent validity by analysing to which degree the EQ-5D correlated with clinical measures of anxiety, depression, and subjective health complaints (De Vet et al., 2015). The tests were carried out using Pearson’s correlation coefficient, a common approach when exploring EQ-5D validity in different patient groups (Byford, 2013; Mulhern et al., 2014). Correlations with the clinical measures were analysed for the EQ-5D values, the EQ VAS, and for all five dimensions. Absolute values larger than +/− 0.50 are considered strong correlations, values between 0.30 and 0.49 moderate, and values beneath 0.30 are considered weak correlations (Fleiss, 1982).

Convergent validity was further explored using a multiple linear regression model. Analyses of multicollinearity were carried out for the explanatory variables in the regression model. No explanatory variable had a VIF higher than 1.58, indicating that multicollinearity was not an issue. The regression model explored the relationship between the EQ-5D values, clinical variables, and sociodemographic variables. We were thus interested in the unique variance contribution of each explanatory variable. Partial correlation was thus calculated for each variable to determine its unique contribution to variance.

## Results

### Participant Characteristics

Table 1 shows characteristics of patients. The average age was 36.8years, and there were more females than males (69.6%). The majority were currently living with a partner, either as cohabiting or married (60.5%). On average the patients had a high level of education, there were 79.5% who had some form of higher education, whilst 20.5% had primary or secondary education. Almost half the patients were on some form of sick leave (45.7%), whilst the rest (54.3%) were fully working with no form of social benefits. Scores on the BDI-II and the BAI indicated moderate levels of depression and anxiety. The most common primary diagnosis was F41.1 Generalised Anxiety Disorder (16.1%), followed by F32.1 Moderate Depressive Disorder (12.6%). Depression disorders accounted for 46.07% and anxiety disorders accounted for 36.07% of the diagnoses in the sample. The most prevalent diagnoses that were not strictly an anxiety or depression disorder were still diagnoses associated with these disorders: F43.2 Adjustment disorder (7.6%), and F41.2 Mixed anxiety and depressed mood (4.4%). Secondary diagnoses were not recorded in the study. The mean EQ-5D value was 0.631 indicating that these patients perceived their health status as reduced compared to “full health” anchored at 1.0 on this measure (Devlin et al., 2020). The mean score of the EQ-5D VAS was 55.7. Floor and ceiling effects were negligible for all self-report questionnaires. There were 10 patients (1.1%) who reported “No problems” on all EQ-5D dimensions, indicating a ceiling effect, no patients responded “Extreme problems” on all dimensions. No patients reported scores indicating a ceiling effect on the BDI-II, the BAI, or the SHC. Three patients (0.3%) reported scores indicating a floor effect on the BDI-II, one patient (0.1%) on the SHC.

**Table 1:** Characteristics of patients (*N*=890).

	*n*	%	Mean	SD	Median	IQR
Gender						
Female	619	69.55				
Male	271	30.45				
Age, years			36.83	10.45	35	28–45
18–30	313	35.17				
31–40	272	30.56				
41–50	189	21.24				
51–60	102	11.46				
61–70	14	1.57				
Cohabiting/married	535	60.45				
Education						
Primary/Secondary	179	20.48				
Higher education ≤4yrs	324	37.07				
Higher education >4yrs	371	42.45				
Employment status						
Sick leave	405	45.66				
Fully working	482	54.34				
Health status						
Anxiety (BAI)			18.74	10.12	18	11–26
Depression (BDI-II)			26.09	8.99	26	20–31
Subjective health (SHC)			23.03	10.17	22	16–29
EQ-5D value			0.631	0.187	0.696	0.501–0.750
EQ VAS			55.7	17.7	60	40–70

BAI, the beck anxiety inventory; BDI-II, the beck depression inventory-II; SHC, subjective health complaints; and IQR, interquartile range.

The proportion of participant responses across domains and by level is reported in Table 2. More than two thirds of the patients (68.9%) reported “moderate” to “extreme” problems on the Anxiety/depression dimension of the EQ-5D. No participants reported the highest level of severity on the Mobility or Self-care dimensions. These two dimensions also had the largest number of patients reporting “No problems,” which was 75.7 and 84.8%, respectively.

**Table 2:** Distribution of all recorded EQ-5D responses in the patient sample (*N*=890).

Severity	Mobility		Self-care		Usual activities		Pain/discomfort		Anxiety/depression	
	*n*	%	*n*	%	*n*	%	*n*	%	*n*	%
1	674	75.7	755	84.8	193	21.7	212	23.8	30	3.4
2	146	16.4	101	11.4	340	38.2	375	42.1	234	26.3
3	43	4.8	17	1.9	237	26.6	229	25.7	363	40.8
4	12	1.4	4	0.5	99	11.1	51	5.7	230	25.8
5					9	1.0	8	0.9	20	2.3

Severity of problems: 1 No problems; 2 Some problems; 3 Moderate problems; 4 Severe problems; and 5 Extreme problems.

The mean EQ-5D values and EQ VAS scores by sociodemographic and clinical characteristics are presented in Table 3.

**Table 3:** Mean EQ-5D value and EQ VAS by sociodemographic and clinical characteristics (*N*=890).

	EQ-5D value	EQ VAS
	Mean (SD)	Mean (SD)
Gender		
Female	0.635 (0.181)	55.5 (17.4)
Male	0.623 (0.201)	56.0 (18.3)
Age		
18–30	0.622 (0.187)	65.9 (18.6)
31–40	0.623 (0.197)	64.6 (18.8)
41–50	0.667 (0.168)	62.9 (19.1)
51–60	0.618 (0.209)	66.2 (19.7)
61–70	0.562 (0.190)	55.7 (16.5)
Cohabitation		
Living with partner	0.633 (0.182)	55.8 (17.6)
Living alone	0.616 (0.189)	55.3 (17.8)
Education		
Primary/secondary	0.586 (0.191)	51.8 (17.5)
Higher education	0.643 (0.184)	56.5 (17.6)
Employment		
Sick leave	0.608 (0.193)	52.0 (18.4)
Fully working	0.651 (0.179)	58.7 (16.4)
Primary diagnosis		
Depression	0.624 (0.184)	53.3 (18.1)
Anxiety	0.625 (0.190)	57.6 (16.9)

Variation in health status by patient characteristics as recorded by the EQ-5D value and the EQ Visual analogue scale (VAS).

### Known-Groups Validity

**Figure 1:**
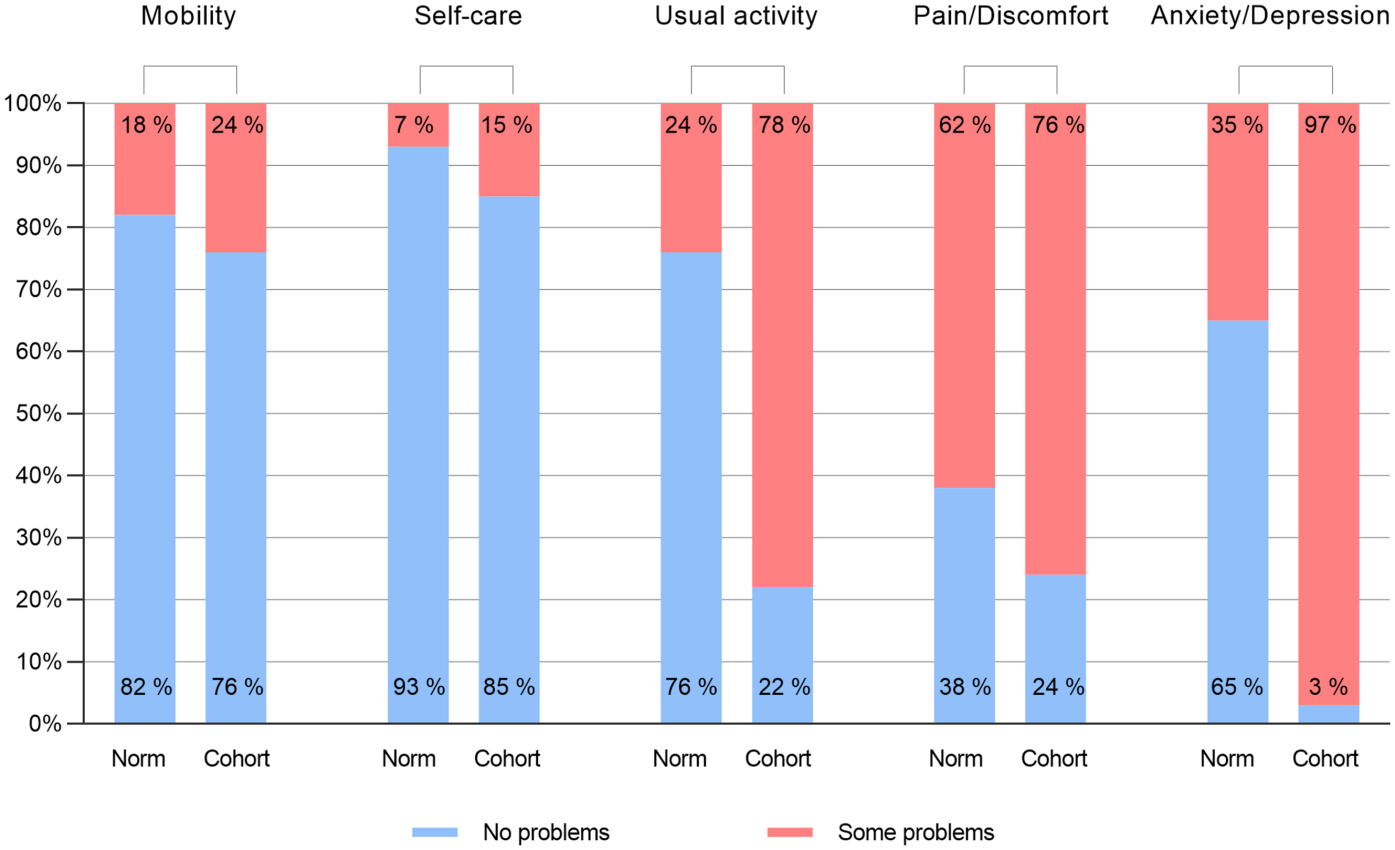
Comparing proportion of EQ-5D “No problems” responses to the Norwegian general population. The “Cohort” columns represent the proportion of patients in the current study who reported “No problems” on each dimension of the EQ-SD compared to the patients who reported any other level of severity. The “Norm” columns represent the same proportion from the study that collected Norwegian population norms; respondents who reported “No problems “on the various dimensions compared to all other levels of severity (Garrat et al., 2021). Percentages are rounded to the nearest integer.

All participants in the study reported that they had at least some problems on EQ-5D. The highest proportion of problems was seen in the Anxiety/depression, where 96.6% of patients reported some level of problems. A minority of patients reported problems on the Mobility and Self-care; 24.3 and 15.2%, respectively, whilst a large majority of patients in the sample reported some level of problems on Usual activities and Pain/discomfort (78.3 and 76.2%, respectively). Figure 1 shows a comparison between the proportion of patients in the present study and the proportion of participants in the study obtaining data for the general population norms (Garratt et al., 2021) reporting “No problems” on the sub-scales. The patient cohort reported more problems on all dimensions compared to the norm population.

Test of Cuzick (1985) for trends showed that there was significant difference between the EQ-5D utility scores when patients were divided into quartiles based on severity of depression and anxiety symptoms, *Z*=− 16.58, *p*=<0.001. As severity of symptoms increased, health as recorded by the EQ-5D utility decreased (Table 4). Similarly, the ordinal logistic regression showed that EQ-5D dimensions significantly predicted the symptom severity groups. All dimensions were significant predictors, while the largest contribution was made by the Anxiety/depression dimension (Table 5).

**Table 4:** Severity of depression and anxiety symptoms by quartiles (*N*=890).

Severity quartile	*n*	BDI-II	BAI	EQ-5D utility
		Median	Median	Median
1	224	17	9	0.767
2	230	24	13	0.721
3	214	28	20	0.689
4	222	35	29	0.476

BAI, the beck anxiety inventory; BDI-II, the beck depression inventory-II.

**Table 5:** Ordinal logistic regression predicting severity of depression and anxiety symptoms (*N*=890).

EQ-5D dimension	OR	SE	*z*	*p*	95% CI	Wald	Prob. *X*^2^	Pseudo *R*^2^
Mobility	1.45	0.170	3.17	0.002	1.15–1.83	381.60	0.000	0.158
Self-care	1.41	0.240	2.03	0.042	1.01–1.97			
Activity	1.49	0.117	5.06	<0.001	1.28–1.73			
Pain	1.77	0.143	7.05	<0.001	1.51–2.07			
Anxiety/depression	2.56	0.227	10.58	<0.001	2.15–3.04			

Severity of depression and anxiety symptoms by quartiles is the dependant variable, and severity of problems reported on the EQ-5D dimensions are the predictor variables.

### Convergent Validity

Pearson’s correlation coefficient showed that the EQ-5D values had a significantly strong negative correlation with the BDI-II depression score, and a moderate negative correlation with the BAI anxiety score and the SHC score. This indicates that for all clinical scales, higher symptom severity correlated with worse reported health status on the EQ-5D value.

For the EQ-5D dimensions, the BDI-II showed a significant moderate correlation with Usual activities and the Anxiety/depression dimension. The BAI showed a significant moderate correlation with Anxiety/depression, whilst SHC showed a significant strong correlation with the Pain/discomfort. Note that lower scores on each dimension indicate better health, i.e., a “1” indicates “no problems.” The moderate to strong correlations in these results thus indicate that lower clinical scores signifying better health were associated with better health reported across the EQ-5D dimensions. Pearsons’s correlation coefficients are presented in Table 6.

**Table 6:** Pearson’s correlation coefficient between the EQ-5D and clinical measures (*N*=890).

	BAI	BDI-II	SHC
EQ-5D value	−0.49	−0.52	−0.44
EQ VAS	−0.27	−0.46	−0.31
EQ-5D dimensions			
Mobility	0.30	0.23	0.23
Self-care	0.15	0.30	0.18
Usual activities	0.24	0.45	0.25
Pain/discomfort	0.38	0.33	0.50
Anxiety/depression	0.42	0.46	0.29

All correlations significant at *p*≤0.001. Correlations below 0.3 are considered weak, above 0.4 moderate, above 0.5 are considered strong (Fleiss, 1982). Note that for the EQ-5D value and VAS, higher scores indicate better health. For the dimensions, lower scores indicate better health. BAI, the beck anxiety inventory; BDI-II, the beck depression inventory-II; and SHC, subjective health complaints.

The multivariate regression model for convergent validity showed that higher levels of depression and anxiety symptoms, more subjective health complaints, being on sick leave, and being female, all significantly predicted lower EQ-5D value, i.e., worse health status, *F*(8, 876)=65.24, *p*<0.000, *R*^2^=0.401. We examined the partial correlation for the variables that were significant predictors in the model: For gender it was *r*=0.13, *p*<0.001; for BDI-II *r*=0.38, *p*<0.001; for BAI *r*=0.28, *p*<0.001; for SHC *r*=0.13, *p*<0.001; and for sick leave *r*=0.09, *p*<0.001. The largest proportion of the variance in the model was thus explained by depression and anxiety, respectively. Results from the regression model is presented in Table 7.

**Table 7:** Regression analysis predicting the EQ-5D value (*N*=890).

	Coef.	*SE*	*T*	*P*	95% CI	Beta coef.	*F*	*R* ^2^	Adj. *R*^2^
Age	0.0004	0.0005	0.77	0.439	−0.0006 to 0.0014	0.0216	73.34	0.406	0.401
Gender	−0.0423	0.0112	−3.78	<0.001	−0.0643 to −0.0203	−0.1047			
Cohabitation	0.0086	0.0102	0.84	0.399	−0.0114 to 0.0287	0.0227			
Education	0.0209	0.0128	1.73	0.085	−0.0030 to 0.0472	0.0481			
BDI-II	−0.0076	0.0006	−11.96	<0.001	−0.0087 to −0.0062	−0.3621			
BAI	−0.0052	0.0006	−8.40	<0.001	−0.0064 to −0.0040	−0.2825			
SHC	−0.0024	0.0006	−3.81	<0.001	−0.0037 to −0.0012	−0.1339			
Sick leave	−0.0274	0.0101	−2.76	0.006	−0.0478 to −0.0080	−0.0749			

EQ-5D value is the dependant variable and demographic characteristics, BDI-II, BAI, SHC and sick leave are predictors. BAI, the beck anxiety inventory; BDI-II, the beck depression inventory-II; and SHC, subjective health complaints.

## Discussion

Our aim was to investigate the validity of the EQ-5D in patients on or at risk of sick leave due to depression and anxiety by examining the health status reported by the EQ-5D. Patients in the study reported poorer health status on the EQ-5D than the normal population. Known-groups validity was supported by both the EQ-5D utility value and the dimensions being able to discriminate between patient groups based on severity of depression and anxiety symptoms. Convergent validity was supported by the EQ-5D showing strong correlations with the BDI-II, and moderate correlation with the BAI and the SHC. Finally, the clinical measures in the study significantly predicted overall health as recorded by the EQ-5D value.

In the current study, all dimensions of the EQ-5D had patients who reported at least some degree of problems. As would be expected in a sample of patients with depression and anxiety diagnoses, highest incidence of problems was reported on the Anxiety/depression dimension. A total of 96.6% of patients reported problems of varying severity on this dimension. The majority of patients also reported problems on the Usual activities and Pain/discomfort dimensions, 78.3 and 76.2%, respectively. This is in line with previous research, which has shown that both functional impairment and pain are prevalent in depression and anxiety (de Heer et al., 2014; McKnight et al., 2016; Hammer-Helmich et al., 2018). A majority of patients reported “No problems” on the Mobility and Self-care dimensions, 75.7 and 84.8%, respectively. We would suggest that this is consistent with the clinical characteristics of the sample. The patients reported moderate levels of depression and/or anxiety, which would not usually entail difficulties with mobility or washing and dressing. Overall, patients in the study reported more problems across all dimensions compared to the respondents in the study that collected the Norwegian EQ-5D norm data (Garratt et al., 2021).

Our findings show that patients experienced reduced health status with a mean EQ-5D value of 0.631 (*SD*=0.187) and a mean EQ VAS score of 55.6 (*SD*=17.7). The EQ-5D value was reduced compared to the “full health” anchoring at 1.0, and also compared to the Norwegian study obtaining population norms which found a mean EQ-5D value of 0.805 and a mean EQ VAS of 77.9 in their postal survey (Garratt et al., 2021). A previous study of Norwegian patients with common mental disorders used the three-level version of the EQ-5D, and reported a mean EQ VAS of 65.6 (Reme et al., 2015). The present study seems to add to this finding and indicates that the EQ-5D as expected reports reduced health status in patients with depression and anxiety when compared to a non-clinical population.

When the patients in the study were divided into quartiles based on severity of depression and anxiety symptoms, and the EQ-5D value reported significantly poorer health with increasing symptom severity. Similarly, the ordinal regression model showed that problems reported on all EQ-5D dimensions increased with symptom severity. The largest contribution to the model was made by the Anxiety/depression dimension, which seems to support validity.

The EQ-5D value showed moderate correlations with the measures of anxiety and subjective health complaints, and strong correlation with the depression measure. The patients in the current study had all been referred to specialised care for treatment of depression and anxiety, and we would thus want to see significant correlations with condition-specific measures to support the validity in this patient group. For the five dimensions of the EQ-5D, the BDI-II and the BAI showed moderate correlations with the Anxiety/depression dimension. The BDI-II also showed a moderate correlation with Usual activities, whilst the BAI only had a weak correlation with this dimension. This reflects previous research which indicates that depression has a clear link to functional impairment, whilst the link to anxiety is more ambiguous (McKnight et al., 2016; Hammer-Helmich et al., 2018). For musculoskeletal complaints, the SHC showed a strong correlation with the Pain/discomfort dimension. That the BDI-II and the BAI both showed the strongest correlation with the Anxiety/depression dimensions, whilst the SHC showed the strongest correlation with the Pain/discomfort dimensions is consistent with discriminant validity as the dimensions provide a differentiated pattern of correlations. The pattern of correlations between the condition-specific measures and the relevant EQ-5D dimensions thus seems to further support convergent validity as the highest correlations are found between conceptually related dimensions and conditions-specific measures.

The final regression analysis indicated that a substantial part of the EQ-5D value was explained by the condition-specific measures in the study. The only significant socioeconomic variable in the study was gender. This finding is consistent with previous research which has shown a gender gap in self-reported health, where women report poorer health than men (Boerma et al., 2016). Women also have generally higher rates of sick leave than men across developed countries, including Norway, where the present study was conducted (Mastekaasa and Melsom, 2014). Although several explanations have been offered, such as a potential extra burden on women as caretakers in the home, the reasons for this gender gap is still poorly understood (Ostby et al., 2018).

That age, education level and cohabitation did not influence health as recorded by the EQ-5D is perhaps more unexpected. Previous research has shown that these factor tend to influence health status (Braveman and Gottlieb, 2014). This is also true when considering health as recorded by the EQ-5D, where age in particular has been shown to influence self-reported health (Stavem et al., 2018). It may be that the sample was too heterogenous to detect differences in the current study. The patients were quite young with a mean age of 36.8years, and most had higher education. Perhaps a more diverse selection of patients would produce different results on this count.

The BDI-II, which measures depression, was the largest predictor in the regression model, followed by anxiety measured by the BAI. Furthermore, SHC and sick leave also made significant contributions, indicating that the EQ-5D value was sensitive to musculoskeletal pain and functional impairment. The second regression model explained 40.1% of the variance of the EQ-5D value. The explanatory variables of the model represent a fairly broad clinical evaluation of patients with depression and anxiety. These variables in turn explained a reasonable proportion of the variance of the EQ-5D value. Furthermore, the largest contributors to explained variance were instruments measuring the severity of these patients’ primary diagnoses. The results of the regression analyses thus suggest that the variation in the EQ-5D value may be a valid proxy for overall health status as it is associated with the variations in severity of the symptoms reported in this patient group.

Finally, it is worth mentioning that the ceiling and floor effects of the EQ-5D were negligible in the study. This indicates that the EQ-5D seems to have had adequate range in capturing health status for these patients. It is a particular interesting aspect as of the first version of the EQ-5D had difficulties with floor and ceiling effects, including for mental health (Herdman et al., 2011). There were also few missing items, less than 2% on all dimensions. This further suggests that the EQ-5D may be a feasible instrument for these patients.

### Implications

The current study suggests that the five-level version of the EQ-5D may be a useful generic measure for evaluating health status in patients on or at risk of sick leave due to depression and anxiety. Including the instrument when assessing burden of disease in these patients may thus facilitate comparison with other patient groups.

Furthermore, functional impairment has emerged as a key component of depression and anxiety. This is especially true of depression, where it also increases risk of relapse (Hardeveld et al., 2010). This functional impairment often manifests as sick leave and work disability, incurring high costs for both individual patients and wider society (OECD, 2015b). This has led to calls for including broader measures of function in evaluating the impact of depression and anxiety on patients (Hardeveld et al., 2010; Chevance et al., 2020). The present study indicates that the EQ-5D may be a valid option to provide a broader measure of health for these patients.

There is also considerable interest in calculating the cost associated with depression and anxiety, and the potential benefits associated with treatment. Multiple studies suggest that better access to treatment would pay for itself, which is one of the key arguments underpinning the UK’s Increasing Access to Psychological Therapies (IAPT) programme (Layard and Clark, 2015). These arguments are often based on broad estimates of increased productivity due to beneficial treatment outcomes (Chisholm et al., 2016). The EQ-5D values may help inform such estimates by providing data from clinical trials supporting cost-effectiveness analyses using QALYs. The calculation of QALYs does however depend on adequately measuring health status over time. Future studies should assess this ability of the EQ-5D in mental health.

### Strengths and Limitations

This is the first study to investigate the validity of the five-level version of the EQ-5D in a large patient cohort on or at risk of sick leave due to depression and anxiety. The study had a large sample size, and patients were screened and diagnosed in a specialised mental health service clinic, providing high-quality measures of clinical characteristics.

In lack of Norwegian tariffs, the recommendation is to use of the EQ-5D UK value set. Recent research demonstrates that choice of value set can have a significant impact on EQ-5D values produced (Camacho et al., 2018). It is therefore necessary to replicate the present findings using a Norwegian tariff in future studies when these are available.

However, the health profile recorded from the EQ-5D questionnaire would remain the same and therefore many of the conclusions of the study are fixed. The study included a varied, but limited, range of clinical measures and sociodemographic. Further research could explore the correlation between the EQ-5D and other types of measures, such as capability measures, and wider determinants of health. The relationship between type of sick leave, and the role of the welfare system is worth considering. The current study did not include information on degree or duration of sick leave. It is also worth mentioning that the Norwegian welfare system is relatively generous compared to many other countries (Andreß and Heien, 2001). Employees receive compensation equivalent to 100% of their salary from the first day of sick leave. This is covered by employers for the first 16days, and then subsequently by the state welfare system for up to a year. It is possible that the relationship between health status recorded by the EQ-5D, and sick leave could vary by country, given the substantial variation between national welfare systems and conditions of sick leave.

The current study also included more women than men. Although this may raise questions of generalisability, the gender distribution reflects the prevalence patterns of mental disorders (Boyd et al., 2015). We also used a binary approach to gender, and we thus do not know whether the study may have included non-binary participants. Finally, the clinical validity explored in the present study is an important psychometric property of an instrument, but it is not the same as clinical responsiveness (Payakachat et al., 2015). Future research should examine to which degree the EQ-5D is responsive to change in health status in mental health patients, for instance in the shape of recovery from depression and anxiety.

## Conclusion

In the present study, the EQ-5D showed evidence of construct validity in patients on or at risk of sick leave due to depression and anxiety. The EQ-5D value was sensitive to both clinical symptoms and to functional impairment in the form of sick leave. The findings thus support the validity of the EQ-5D as a measure of health status for these patients. These results need to be replicated in other samples and different sociodemographic settings. However, the current findings suggests that the EQ-5D is a feasible instrument when evaluating health status of patients of patients with depression and anxiety.

## Data Availability Statement

The data used in the article is not readily available as the participants have not consented to distribution beyond the use in the study. Requests to access the datasets should be directed to Kenneth Sandin, kenneth.sandin@diakonsyk.no.

## Ethics Statement

Ethical review and approval was not required for the study on human participants in accordance with the local legislation and institutional requirements. The patients/participants provided their written informed consent to participate in this study.

## Author Contributions

KS led the writing of the manuscript and is the principal author of the funding application. GS contributed to conceptualisation, data analyses, and writing. RG is the clinic leader and contributed to conceptualisation and writing. KO was responsible for management and structuring of data. MB contributed to data structuring and writing. OH is the project manager and contributed to design, analyses, and writing. All authors contributed to the article and approved the submitted version.

## Funding

The study was mainly sponsored by Diakonhjemmet Hospital with additional funding from Southern and Eastern Norway Regional Health Authority, and Stiftelsen Dam (previously Extrastiftelsen), a not for profit trust that funds health research and innovation projects.

## Conflict of Interest

The authors declare that the research was conducted in the absence of any commercial or financial relationships that could be construed as a potential conflict of interest.

## Publisher’s Note

All claims expressed in this article are solely those of the authors and do not necessarily represent those of their affiliated organizations, or those of the publisher, the editors and the reviewers. Any product that may be evaluated in this article, or claim that may be made by its manufacturer, is not guaranteed or endorsed by the publisher.

## References

[ref1] Andreß H. J. Heien T. (2001). Four worlds of welfare state attitudes? A comparison of Germany, Norway, and the United States. Eur. Sociol. Rev. 17, 337–356. doi: 10.1093/esr/17.4.337

[ref2] Asmundson G. J. Katz J. (2009). Understanding the co-occurrence of anxiety disorders and chronic pain: state-of-the-art. Depress. Anxiety 26, 888–901. doi: 10.1002/da.20600, PMID: 19691031

[ref3] Bair M. Robinsons R. L. Katon W. Kroenke K. (2003). Depression and pain comorbidity: a literature review. Arch. Intern. Med. 163, 2433–2445. doi: 10.1001/archinte.163.20.2433, PMID: 14609780

[ref4] Beck A. T. Epstein N. Brown G. K. Steer R. A. (1988). An inventory for measuring clinical anxiety: psychometric properties. J. Consult. Clin. Psychol. 56, 893–897. doi: 10.1037/0022-006X.56.6.893, PMID: 3204199

[ref5] Beck A. T. Steer R. A. Brown G. K. (1996). Manual for Beck Depression Inventory II (BDI-II). San Antonio: Psychology Corporation.

[ref6] Boerma T. Hosseinpoor A. R. Verdes E. Chatterji S. (2016). A global assessment of the gender gap in self-reported health with survey data from 59 countries. BMC Public Health 16:675. doi: 10.1186/s12889-016-3352-y, PMID: 27475755 PMC4967305

[ref7] Boyd A. Van de Velde S. Vilagut G. de Graaf R. O’Neill S. Florescu S.. (2015). Gender differences in mental disorders and suicidality in Europe: results from a large cross-sectional population-based study. J. Affect. Disord. 1, 245–254. doi: 10.1016/j.jad.2014.11.002 25462424

[ref8] Braveman P. Gottlieb L. (2014). The social determinants of health: it’s time to consider the causes of the causes. Public Health Rep. 129, 19–31. doi: 10.1177/00333549141291S206, PMID: 24385661 PMC3863696

[ref9] Brazier J. Connell J. Papaioannou D. Mukuria C. Mulhern B. Peasgood T.. (2014). A systematic review, psychometric analysis and qualitative assessment of generic preference-based measures of health in mental health populations and the estimation of mapping functions from widely used specific measures. Health Technol. Assess. 18, 1–188. doi: 10.3310/hta18340, PMID: 24857402 PMC4781324

[ref10] Byford S. (2013). The validity and responsiveness of the EQ-5D measure of health-related quality of life in an adolescent population with persistent major depression. J. Ment. Health 22, 101–110. doi: 10.3109/09638237.2013.779366, PMID: 23574502

[ref11] Camacho E. M. Shields G. Lovell K. Coventry P. A. Morrison A. P. Davies L. M. (2018). A (five-)level playing field for mental health conditions?: exploratory analysis of EQ-5D-5L-derived utility values. Qual. Life Res. 27, 717–724. doi: 10.1007/s11136-017-1768-1, PMID: 29248995 PMC5845602

[ref12] Chevance A. Ravaud P. Tomlinson A. Le Berre C. Teufer B. Touboul S.. (2020). Identifying outcomes for depression that matter to patients, informal caregivers, and health-care professionals: qualitative content analysis of a large international online survey. Lancet Psychiatry 7, 692–702. doi: 10.1016/S2215-0366(20)30191-7, PMID: 32711710

[ref13] Chin C. L. Yao G. (2014). “Convergent Validity,” in Encyclopedia of Quality of Life and Well-Being Research. ed. Michalos A. (Dordrecht: Springer)

[ref14] Chisholm D. Sweeny K. Sheehan P. Rasmussen B. Smit F. Cuijpers P.. (2016). Scaling-up treatment of depression and anxiety: a global return on investment analysis. Lancet Psychiatry 3, 415–424. doi: 10.1016/S2215-0366(16)30024-4, PMID: 27083119

[ref15] Cullen K. Irvin E. Collie A. Clay F. Gensby U. Jennings P.. (2018). Effectiveness of workplace interventions in return-to-work for musculoskeletal, pain-related and mental health conditions: an update of the evidence and messages for practitioners. J. Occup. Rehabil. 28, 1–15. doi: 10.1007/s10926-016-9690-x, PMID: 28224415 PMC5820404

[ref16] Cuzick J. (1985). A wilcoxon-type test for trends. Stat. Med. 4, 87–90. doi: 10.1002/sim.4780040112, PMID: 3992076

[ref17] Davidson M. (2014). “Known-group validity,” in Encyclopedia of Quality of Life and Well-Being Research. ed. Michalos A. (Dordrecht: Springer)

[ref18] de Heer E. W. Gerrits M. M. J. G. Beekman A. T. F. Dekker J. van Marwijk H. W. J. de Waal M. W. M.. (2014). The association of depression and anxiety with pain: a study from NESDA. PLoS One 9:e106907. doi: 10.1371/journal.pone.0106907, PMID: 25330004 PMC4198088

[ref19] De Vet H. C. W. Terwee C. B. Mokkink L. B. Knol D. L. (2015). Measurement in Medicine. 5th *Edn*. Cambridge: Cambridge University Press.

[ref20] de Vries H. Fishta A. Weikert B. Rodriguez Sanchez A. Wegewitz U. (2018). Determinants of sickness absence and return to work among employees with common mental disorders: a scoping review. J. Occup. Rehabil. 28, 393–417. doi: 10.1007/s10926-017-9730-1, PMID: 28980107 PMC6096498

[ref21] Devlin N. J. Brooks R. (2017). EQ-5D and the EuroQol group: past, present and future. Appl. Health Econ. Health Policy 15, 127–137. doi: 10.1007/s40258-017-0310-5, PMID: 28194657 PMC5343080

[ref22] Devlin N. J. Parkin D. Janssen B. (2020). Methods for Analysing and Reporting EQ-5D Data. Springer International Publishing. 33347096

[ref23] Drummond M. F. Schulper M. J. Claxton K. Stoddart G. L. Torrance G. W. (2015). Methods for the Economic Evaluation of Health Care Programmes. 4th *Edn*. Oxford: Oxford University Press.

[ref24] Eriksen H. R. Ihlebaek C. Ursin H. A. (1999). A scoring system for subjective health complaints (SHC). Scand. J. Public Health 27, 63–72. doi: 10.1177/14034948990270010401, PMID: 10847674

[ref25] Eriksson H. G. von Celsing A. S. Wahlstrom R. Janson L. Zander V. Wallman T. (2008). Sickness absence and self-reported health a population-based study of 43,600 individuals in central Sweden. BMC Public Health 8:426. doi: 10.1186/1471-2458-8-426, PMID: 19116000 PMC2627845

[ref26] EuroQol (1990). A new facility for the measurement of health-related quality of life. Health Policy 16, 199–208. doi: 10.1016/0168-8510(90)90421-9, PMID: 10109801

[ref27] Fleiss J. L. (1982). Statistical Methods for Rates and Proportions. New York: Wiley & Sons.

[ref28] Gale T. Hawley C. (2001). A model for handling missing items on two depression rating scales. Int. Clin. Psychopharmacol. 16, 205–214. doi: 10.1097/00004850-200107000-00004, PMID: 11459334

[ref29] Garratt A. M. Hansen T. M. Augestad L. A. Rand K. Stavem K. (2021). Norwegian population norms for the EQ-5D-5L: results from a general population survey. Qual. Life Res. 1–10. doi: 10.1007/s11136-021-02938-7, PMID: 34272631 PMC8284681

[ref30] Gjengedal R. G. H. Reme S. Osnes K. Lagerveld S. Blonk R. Sandin K.. (2020). Work-focused treatment for common mental disorders: an observational study comparing an intervention group with a waiting list control group. Work 6, 657–667. doi: 10.3233/WOR-203208 PMC7504991 32623425

[ref31] Hair J. F. Hult G. T. M. Ringle C. M. Sarstedt M. (2017). A Primer on Partial Least Squares Structural Equation Modeling (PLS-SEM). 2nd *Edn*. Thousand Oaks, CA: Sage.

[ref32] Hammer-Helmich L. Haro J. M. Jonsson B. Melac A. T. Di Nicola S. Chollet J.. (2018). Functional impairment in patients with major depressive disorder: the 2-year PERFORM study. Neuropsychiatr. Dis. Treat. 14, 239–249. doi: 10.2147/NDT.S146098, PMID: 29386897 PMC5767094

[ref33] Hardeveld F. Spijker J. De Graaf R. Nolen W. A. Beekman A. T. (2010). Prevalence and predictors of recurrence of major depressive disorder in the adult population. Acta Psychiatr. Scand. 122, 184–191. doi: 10.1111/j.1600-0447.2009.01519.x, PMID: 20003092

[ref34] Herdman M. Gudex C. Lloyd A. Janssen M. Kind P. Parkin D.. (2011). Development and preliminary testing of the new five-level version of EQ-5D (EQ-5D-5L). Qual. Life Res. 20, 1727–1736. doi: 10.1007/s11136-011-9903-x, PMID: 21479777 PMC3220807

[ref35] Jakobsen J. C. Gluud C. Wetterslev J. Winkel P. (2017). When and how should multiple imputation be used for handling missing data in randomised clinical trials—a practical guide with flowcharts. BMC Med. Res. Methodol. 17:162. doi: 10.1186/s12874-017-0442-1, PMID: 29207961 PMC5717805

[ref36] Lamers F. van Oppen P. Comijs H. C. Smit J. H. Spinhoven P. van Balkom A. J. L. M.. (2011). Comorbitiy patterns of anxiety and depressive disorders in a large cohort study: the Netherlands study of depression and anxity (NESDA). J. Clin. Psychiatry 72, 341–348. doi: 10.4088/JCP.10m06176blu, PMID: 21294994

[ref37] Layard R. Clark D. M. (2015). Why more psychological therapy would cost nothing. Front. Psychol. 6:1713. doi: 10.3389/fpsyg.2015.01713, PMID: 26635648 PMC4658447

[ref38] Lecrubier T. Sheehan D. V. Weiller E. Amorim P. Bonora I. Sheehan K. H.. (1997). The mini international neuropsychiatric interview (MINI). A short diagnostic structured interview: reliability and validity according to the CIDI. Eur. Psychiatry 12, 224–231. doi: 10.1016/S0924-9338(97)83296-8

[ref39] Mastekaasa A. Melsom A. M. (2014). Occupational segregation and gender differences in sickness absence: evidence from 17 European countries. Eur. Sociol. Rev. 30, 582–594. doi: 10.1093/esr/jcu059

[ref40] McKnight P. E. Monfort S. S. Kashdan T. B. Blalock D. V. Calton J. M. (2016). Anxiety symptoms and functional impairment: a systematic review of the correlation between the two measures. Clin. Psychol. Rev. 45, 115–130. doi: 10.1016/j.cpr.2015.10.005, PMID: 26953005

[ref41] Moen Hansen T. Helland Y. Augestad L. A. Rand K. Stavem K. Garratt A. (2020). Elicitation of Norwegian EQ-5D-5L values for hypothetical and experience-based health states based on the EuroQol valuation technology (EQ-VT) protocol. BMJ Open 10:e034683. doi: 10.1136/bmjopen-2019-034683, PMID: 32532768 PMC7295408

[ref42] Mulhern B. Mukuria C. Barkham M. Knapp M. Byford S. Soeteman D.. (2014). Using generic preference-based measures in mental health: psychometric validity of the EQ-5D and SF-6D. Br. J. Psychiatry 205, 236–243. doi: 10.1192/bjp.bp.112.122283, PMID: 24855127

[ref43] NICE (2018). The National Institute of Health and Care Excellence—Guide to the Processes of Technology Appraisal NICE Process and Methods Guides. London: NICE. 27905710

[ref44] NICE (2019). The National Institute of Health and Care Excellence—Position statement on use of the EQ-5D-5L value set for England. Available at: https://www.nice.org.uk/about/what-we-do/our-programmes/nice-guidance/technology-appraisal-guidance/eq-5d-5l (Accessed Jan 10, 2020).

[ref45] NIPH (2019). National Institue of Public Health—Fremskaffing av EQ-5D vekter og normative data for helseøkonomiske evalueringer—prosjektbeskrivelse. (English: Acquisition of EQ-5D weights and normative data for health economic evaluations—project description). Available at: https://www.fhi.no/cristin-prosjekter/aktiv/fremskaffing-av-eq-5d-vekter-og-normative-data-for-helseokonomiske-evalueri/ (Accessed September 27, 2020).

[ref46] Norstrom F. Waenerlund A. K. Lindholm L. Nygren R. Sahlen K. G. Brydsten A. (2019). Does unemployment contribute to poorer health-related quality of life among Swedish adults? BMC Public Health 19:457. doi: 10.1186/s12889-019-6825-y, PMID: 31035994 PMC6489216

[ref47] OECD (2012). Sick on the Job? Myths and Realities About Mental Health and Work. Paris: OECD Publishing.

[ref48] OECD (2015a). Fit Mind, Fit Job: From Evidence to Practice in Mental Health and Work. Paris: OECD Publishing.

[ref49] OECD (2015b). Mental Health and Work: Fit Mind, Fit Job: From Evidence to Practice in Mental Health and Work. Paris: OECD Publishing.

[ref50] OECD/EU (2018). Health at a Glance: Europe 2018: State of Health in the EU Cycle. Paris: OECD Publishing.

[ref51] Ostby K. A. Mykletun A. Nilsen W. (2018). Explaining the gender gap in sickness absence. Occup. Med. 68, 320–326. doi: 10.1093/occmed/kqy062, PMID: 29672758

[ref52] Payakachat N. Ali M. M. Tilford J. M. (2015). Can the EQ-5D detect meaningful change? A systematic review. Pharmacoeconomics 33, 1137–1154. doi: 10.1007/s40273-015-0295-6, PMID: 26040242 PMC4609224

[ref53] Peters G. J. Y. (2014). The alpha and the omega of scale reliability and validity. Eur. Health Psychol. 16. [Preprint]. doi: 10.31234/osf.io/h47fv

[ref54] Piedmont R. L. (2014). “Construct validity,” in Encyclopedia of Quality of Life and Well-Being Research. ed. Michalos A. (Dordecht: Springer)

[ref55] Reme S. E. Grasdal A. L. Lovvik C. Lie S. A. Overland S. (2015). Work-focused cognitive–behavioural therapy and individual job support to increase work participation in common mental disorders: a randomised controlled multicentre trial. Occup. Environ. Med. 72, 745–752. doi: 10.1136/oemed-2014-102700, PMID: 26251065 PMC4602235

[ref56] Salomonsson S. Hedman-Lagerlof E. Ost L. G. (2018). Sickness absence: a systematic review and meta-analysis of psychological treatments for individuals on sick leave due to common mental disorders. Psychol. Med. 48, 1954–1965. doi: 10.1017/S0033291718000065, PMID: 29380722

[ref57] Sobocki P. Ekman M. Ågren H. Krakau I. Runeson B. Mårtensson B.. (2007). Health-related quality of life measured with EQ-5D in patients treated for depression in primary care. Value Health 10, 153–160. doi: 10.1111/j.1524-4733.2006.00162.x, PMID: 17391424

[ref58] Sonntag M. Konnopka A. Leichsenring F. Salzer S. Beutel M. E. Herpertz S.. (2013). Reliability, validity and responsiveness of the EQ-5D in assessing and valuing health status in patients with social phobia. Health Qual. Life Outcomes 11:215. doi: 10.1186/1477-7525-11-215, PMID: 24365384 PMC3878044

[ref59] StataCorp. (2019). Stata Statistical Software: Release 16. College Station, TX.: StataCorp LLC.

[ref60] Stavem K. Augestad L. A. Kristiansen I. S. Rand K. (2018). General population norms for the EQ-5D-3 L in Norway: comparison of postal and web surveys. Health Qual. Life Outcomes 16:204. doi: 10.1186/s12955-018-1029-1, PMID: 30340499 PMC6194590

[ref61] Stigmar K. G. E. Petersson I. F. Jöud A. Grahn B. E. M. (2013). Promoting work ability in a structured national rehabilitation program in patients with musculoskeletal disorders: outcomes and predictors in a prospective cohort study. BMC Musculoskelet. Disord. 14:57. doi: 10.1186/1471-2474-14-57, PMID: 23384339 PMC3626929

[ref62] Vigo D. Thornicroft G. Atun R. (2016). Estimating the true global burden of mental illness. Lancet Psychiatry 3, 171–178. doi: 10.1016/S2215-0366(15)00505-2, PMID: 26851330

[ref63] Voss M. Nylen L. Floderus B. Diderichsen F. Terry P. D. (2004). Unemployment and early cause-specific mortality: a study based on the Swedish twin registry. Am. J. Public Health 94, 2155–2161. doi: 10.2105/AJPH.94.12.2155, PMID: 15569968 PMC1448606

[ref64] Wang Y. P. Gorenstein C. (2013). Psychometric properties of the beck depression inventory-II: a comprehensive review. Braz. J. Psychiatry 35, 416–431. doi: 10.1590/1516-4446-2012-1048, PMID: 24402217

